# Reproductive Output and Insect Behavior in Hybrids and Apomicts from *Limonium ovalifolium* and *L. binervosum* Complexes (Plumbaginaceae) in an Open Cross-Pollination Experiment

**DOI:** 10.3390/plants10010169

**Published:** 2021-01-17

**Authors:** Sofia I. R. Conceição, Joana Fernandes, Elsa Borges da Silva, Ana D. Caperta

**Affiliations:** 1 Linking Landscape, Environment, Agriculture and Food (LEAF), Instituto Superior de Agronomia (ISA), Universidade de Lisboa, Tapada da Ajuda, 1349-017 Lisboa, Portugal; sofia-conceicao@hotmail.com (S.I.R.C.); joanabnf@hotmail.com (J.F.); 2 Forest Research Centre (CEF), Instituto Superior de Agronomia (ISA), Universidade de Lisboa, Tapada da Ajuda, 1349-017 Lisboa, Portugal; elsasilva@isa.ulisboa.pt

**Keywords:** ancillary pollen and stigma polymorphisms, apomixis, flower heteromorphism, pleiocotyly, polyembryony, polyploidy, reproduction

## Abstract

Ex situ plant collections established from seeds of natural populations are key tools for understanding mating systems of intricate taxonomic complexes, as in the *Limonium* Mill. genus (sea lavenders, Plumbaginaceae). Plants show a polymorphic sexual system associated to flower polymorphisms such as ancillary pollen and stigma and/or heterostyly that prevents self and intramorph mating. The main objectives of this study were to investigate the significance of pollen-stigma dimorphisms and the role of flower visitors in the reproductive output of hybrids arising from sexual diploids of *Limonium ovalifolium* complex and apomicts tetraploids of *L. binervosum* complex in an open cross-pollination experiment. Results showed that, similarly to parental plants, hybrids present inflorescence types, self-incompatible flowers, and produced regular pollen grains with the typical exine patterns, with medium to high viability. By contrast, apomicts show floral polymorphisms, inflorescences, and pollen grains of maternal phenotype but with low stainability. Several insects’ species visited the inflorescences of parental plants and both hybrids and apomicts and some of these insects carried A and/or B pollen grains on their bodies, especially *Clepsis coriacana* (Rebel) and *Tapinoma* sp. Insects’ floral visits to hybrids and apomicts seem to be independent of pollen fertility and plants’ reproductive modes. Both hybrids and apomicts were able to produce fertile seeds, although the latter showed more seedlings with developmental anomalies than the first plants. The findings demonstrate that there is a weak reproductive barrier between the diploid species of *L. ovalifolium* complex as they can hybridize and produce fertile hybrids, provided there is pollen transport by pollinator insects. This study supports that apomixis is a strong reproductive barrier between both *L. ovalifolium* and *L. binervosum* complexes but did not allow us to exclude reproductive interferences of apomict pollen into sexuals.

## 1. Introduction

In flowering plants hybridization and polyploidy are important evolutionary processes often connected to changes in the reproductive systems [1,2,3,4]. In the genus *Limonium* Mill. (sea lavenders Plumbaginaceae), plants characteristically show striking flower polymorphisms linked to a sporophytic self-incompatibility system, which is associated with distinct reproductive modes, sexual and/or apomixis (agamospermy, asexual reproduction through seeds) [1,2,3,4,5,6,7,8]. Most species show ancillary pollen (e.g., differences in pollen production, size, shape, and exine sculpturing) and stigma heteromorphisms (e.g., papillae size and morphology) generally linked with heterostyly (pistils’ and stamens’ sizes in alternate morphs) that prevents self and intramorph mating [1,2,3,5,7,8,9]. Some *Limonium* species are heterostylous (e.g., *Limonium vulgare*) [2,3,9,10,11], despite most of them being non-heterostylous but with pollen-stigma polymorphisms [2,7,8,9,12].

**Figure 1:**
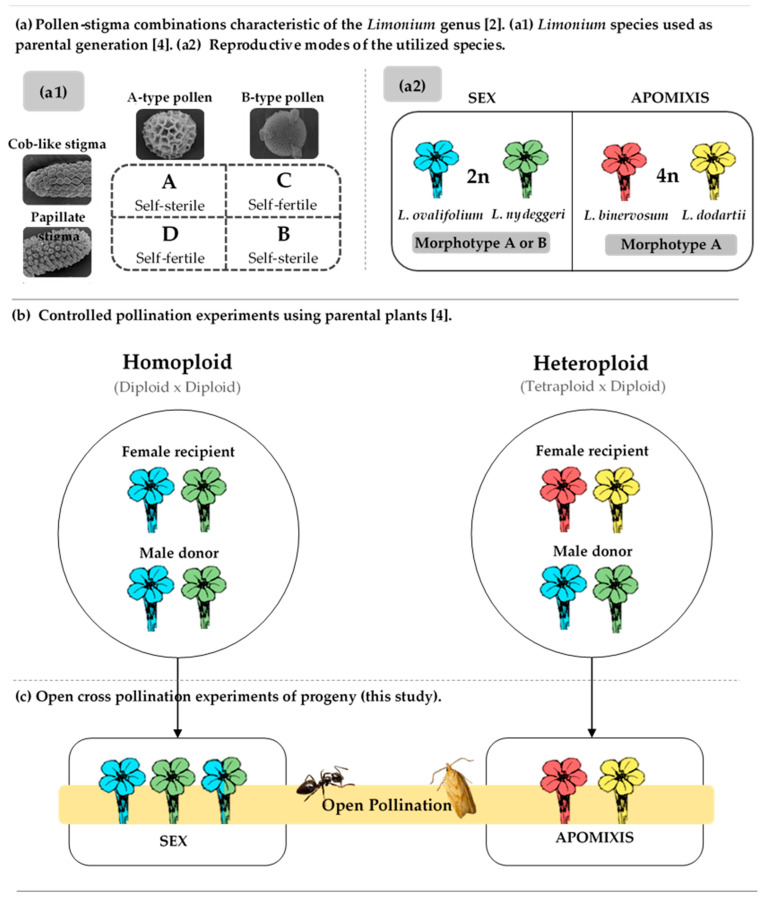
Workflow of the homoploid and heteroploid crosses performed in *Limonium* plants from a controlled ex situ crossing experiment, adapted from [4,7]. For schematic purposes and easy understanding of this figure, the meaning of the color code used for the flowers in section A is maintained throughout the remaining figure sections. In section (**a**), the pollen (A and B) and stigma types (*cob*-like and papillate) found in the *Limonium* genus [7] are represented and exemplified by *L. binervosum*, *L. dodartii*, *L. nydeggeri*, and *L. ovalifolium*. Pollen grains with macroreticulate exine germinate on papillose stigmas surfaces, whereas pollen grains with microreticulate exine germinate on *cob*-like stigmas [1]. Section (**b**) shows the parental species previously characterized in [7] and utilized in the controlled homoploid (diploid × diploid) and heteroploid (tetraploid × diploid) crosses realized in spring 2016 [4] as well as female recipients and male donors used in each cross. The resulting offspring plants were characterized in terms of morphology, ploidy levels, and reproductive modes as assessed by nuclear ribosomal DNA analyses in 2017–18 [4,7]. Section (**c**) represents the plants reproductively characterized in this study. These plants were subjected to open pollination and allowed to be visited by insects in 2019 within a semi-closed greenhouse.

Dimorphic species usually show reciprocal heteromorphic incompatibility, in which flowers of one mating morph produce coarse reticulate sexine (A type pollen) and *cob*-like stigmatic papillae, whereas the complimentary morph shows finely reticulate sexine (B type pollen) and papillate stigmas [1,5,6] (Figure 1a). Dimorphic species’ populations have roughly equal numbers of self-sterile A/*cob* and B/papillate plants and reproduce sexually through outcrossing, as is the case of *Limonium ovalifolium* (Poir.) O. Kuntze and *Limonium nydeggeri* Erben diploid species (2*n* = 2x = 15, 16, 17 chromosomes, most frequent 16) [1,5,6]. In this system, coarsely reticulate pollen grains germinate on papillose stigmas (papillate cells) and finely reticulate grains germinate on *cob*-like stigmas (polygonal cells), while the reverse combinations produce no successful fertilization [1,5,6] (Figure 1a). Monomorphic self-compatible species present A/papillate (D, self-fertile) or B/*cob* combinations (C, self-fertile, more frequent). In contrast, monomorphic self-incompatible populations of species showing only one pollen-stigma combination (either A/cob or B/papillate) seem to produce seeds through apomixis as seen for *Limonium binervosum* (Sm.) C.E. Salmon and *Limonium dodartii* (Girard) Kuntze tetraploid species (2*n* = 4x = 35, 36 chromosomes, most frequent 36) [1,2,3,5,6,7,8] (Figure 1a).

The primary reproductive strategies of *Limonium* species have been inferred upon analyses of such flower heteromorphisms [1,5,6,8]. Generally, sexual species like those of *L. ovalifolium* complex present full anthers (>100 pollen grains, ~ 53.52 ± 5.6 µm) and pollen with high viability (~80–100%; [2,3,7,12]). By contrast, apomicts of the *L. binervosum* complex show empty anthers or few pollen grains (<100), with diverse morphology and sizes [7,12], with high to low fertility, since grains can be partly or totally aborted [2,7,12,13]. Thus, this polymorphic sexual system linked to floral traits (ancillary pollen and stigma polymorphisms) can be considered a prepollination reproductive barrier that separates the diploid and the tetraploid species’ complexes. Both *Limonium* heterostylous sexual [3,5,6,11,14] and apomictic [15] taxa seem to provide floral resources to insect visitors under field conditions. However, whether pollinators have different pollen transfer proficiencies in diploid and tetraploid plants with distinct reproductive modes is unknown.

Molecular genetic studies show evidence for hybrid origin of apomictic taxa (e.g., [4,16,17,18,19,20]) toward vegetative apomixis or agamospermy, as hypothesized for *Limonium* [2,13]. Sexual species like those of diploid *Limonium ovalifolium* complex (*L. ovalifolium*, *L. nydeggeri*, *L. lanceolatum* (Hoffmanns and Link) Franco) mostly form meiotically reduced tetrasporic embryo sacs of *Gagea ova* type and tetrasporous gametophytes of *Adoxa* and *Drusa* types [7,21]. Apomicts such as those of *Limonium binervosum* complex (i.e., *L. binervosum*, *L. dodartii*, *L. multiflorum* Erben) may form meiotically unreduced diplosporous and reduced embryo sacs (facultative apomixis) or unreduced diplosporous embryo sacs and autonomous endosperm (obligate apomicts) [7,22,23]. A previous study using some of those *Limonium* plants in homoploid (diploid × diploid) and heteroploid (tetraploid × diploid) experimental crosses revealed genetically diverse offspring arising from sexual reproduction, whereas maternal, clonal offspring was produced by apomixis [4] (Figure 1).

This study on plants produced in homoploid and heteroploid experimental crosses [4] through sexual or apomictic reproductive modes addressed the following specific questions: (1) Do floral combinations (ancillary pollen and stigma polymorphisms) of parental plants used in the crosses persist in their progeny? (2) Is there a pollinator preference for a flower type? (3) Are there differences between plants produced through different modes of reproduction in terms of reproductive outcomes? To address these questions, we analyzed *Limonium* diploid hybrids and tetraploid apomicts obtained after controlled hand pollinations comparing the reproductive outputs among plants.

## 2. Results

### 2.1. Inflorescence Phenotype, Pollen-Stigma Combinations, and Pollen Viability

In total, 36 plants originated in homoploid crosses and 15 plants produced from heteroploid crosses were analyzed (Figure 1, Table 1). There were significant differences between the two reproductive modes relatively to pollen-stigma combinations (Table 2). Plants originated from intraspecific crosses presented the pollen-stigma dimorphisms and inflorescence morphotypes like both parental plants. Hybrid plants produced in interspecific homoploid crosses using *L. nydeggeri* and *L. ovalifolium* showed either A or B self-incompatible combinations (cross two: five plants with combination B and four plants with combination A; cross three: all plants with combination A; cross five: four plants with combination B and three plants with combination A). Contrastingly, apomicts had always the same self-incompatible pollen-stigma combination of the maternal type (A), except for one plant that showed a self-compatible combination (C) (Table 1). No individuals with self-compatible combination D (microreticulate pollen/cob-like stigma) were found.

These hybrids presented inflorescence morphotypes found in one of the parental plants or of intermediate type, between *L. nydeggeri* and *L. ovalifolium* (Table 1). The plants with intermediate inflorescence (from crosses three and five) showed pollen-stigma combination A or B. Two of the hybrid plants had an inflorescence morphology resembling that of *L. binervosum* (scape: slightly zigzagging, branching dichotomously in the upper third). Plants produced in heteroploid crosses only showed the maternal inflorescence types like apomict tetraploids *L. binervosum* or *L. dodartii*.

The hybrids produced variable numbers of pollen grains per anther with regular roundish morphology and the typical exine patterns, with medium to high fertility (Supplementary Materials Figure S1), mainly with three *colpi* (Table 3) like parental plants [4]. However, in one of the interspecific crosses plants formed pollen grains with atypical *colpi* number, most of them inviable (Table 3). There were significant differences between the two reproductive modes in relation to pollen *colpi* number (Table 2). The apomicts produced roundish pollen grains with the characteristic exine patterns, but with a variable number of *colpi* (from one to five) (Table 3). Although most grains showed regular form and aperture numbers (three *colpi*), these grains presented low pollen fertility (Table 3).

### 2.2. Floral Visitors

**Figure 2:**
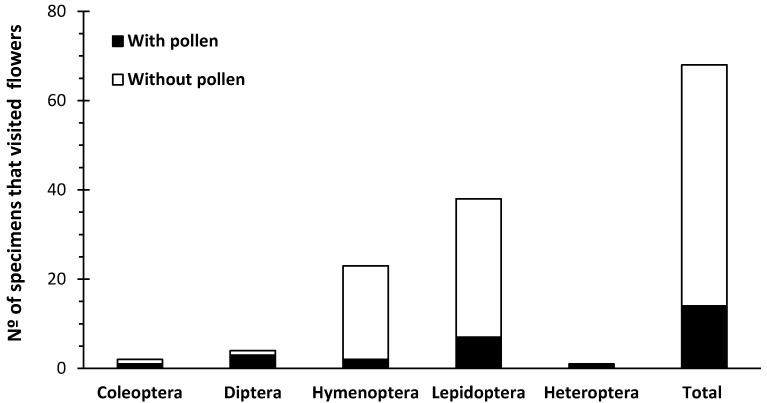
Representativeness of the insects (orders) carrying pollen or not that visited *Limonium* inflorescences.

**Figure 3:**
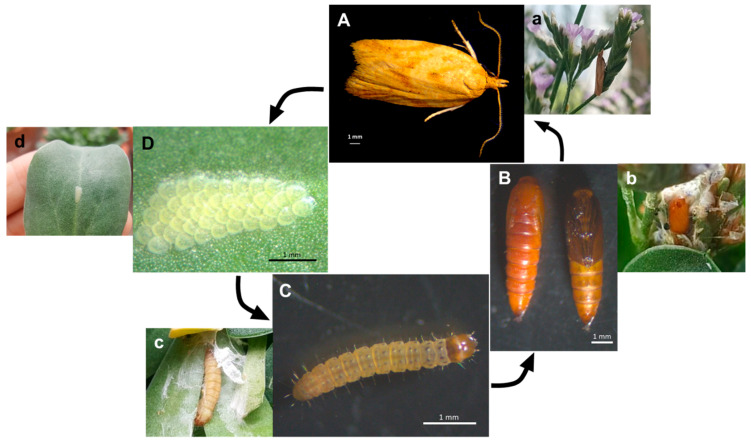
Life cycle of *Clepsis coriacana* (Rebel) (Lepidoptera: Tortricidae) in *Limonium* inflorescences and leaves. (**A**) Male and female adults (**a**); (**B**) pupae and detail of a pupae (**b**); (**C**) larvae and larvae on leaves (**c**); (**D**) eggs’ cluster and eggs’ cluster on a leaf (**d**).

**Figure 4:**
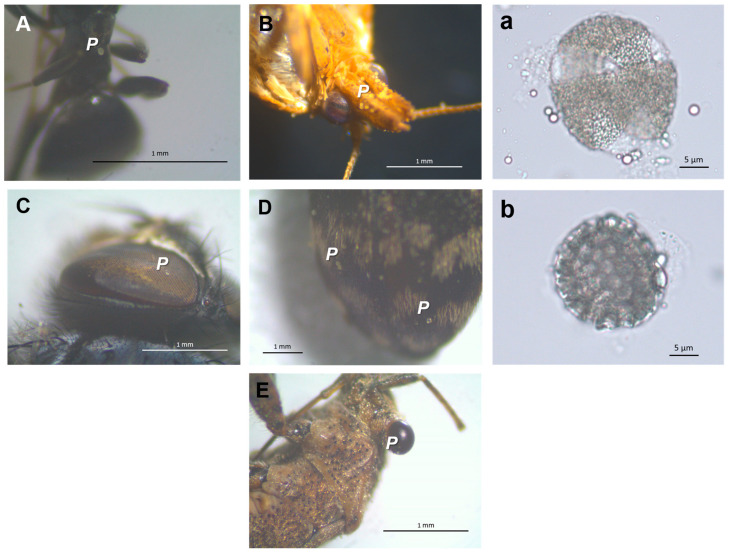
Pollen grains in insect pollinators from the five different orders observed during this study (**A**–**E**). Some of the pollen grains present on each insect captured are circled in red. (**A**) *Tapinoma* sp. (70×); (**B**) *Clepsis coriacana* (40×); (**C**) Diptera (45×); (**D**) Coleoptera (45×); (**E**) Heteroptera (45×); (**a**) *Limonium* pollen grain from A (**a**, *colpi* arrowed) or (**B**) (**b**) types.

In a total of 20 h of observation, 39 specimens of insects visited the inflorescences of parental plants, with no behavioral distinction of preference for either hybrid or apomict plants. The observed insects were from the orders Lepidoptera (55.9%), Diptera (5.9%), Hymenoptera (33.8%), Coleoptera (2.9%), and Heteroptera (1.5%) (Figure 2). Most floral visitors from Hymenoptera were ants of *Tapinoma* sp. (Hymenoptera, Formicidae). *Clepsis coriacana* (Rebel) (Lepidoptera, Tortricidae) was the only Lepidoptera species found in inflorescences and leaves from all plant species (Figure 3). In addition to the behavioral evidence of insects’ visits to the inflorescences, various specimens held pollen grains on their bodies (Figure 4), especially *C. coriacana* (18% with pollen) and *Tapinoma* sp. (9% with pollen). Pollen grains of different sources were identified on the insects’ bodies, including ones *Limonium* A and/or B type pollen (Figure 4). Further, *C. coriacana* individuals in all developmental stages (egg, larvae, pupae, adult) were found on plant structures (Figure 3), including a high level of *C. coriacana exuviae* in stored seeds.

### 2.3. Seed Germination, Seedling Morphotype, and Ploidy Analyses

Plants originated from different reproductive modes presented significant differences in relation to seedling morphotype (Table 2). The hybrids produced variable numbers of seeds per inflorescence, and a few plants of some crosses that showed inflorescences did not produced seeds (e.g., in cross 1 only one plant produced seeds) (Table 4). Plants that did not produce seeds were from both intra- and interspecific crosses and had either combination type A or B. The number of seeds produced by the hybrids was smaller than the tetraploid apomicts (Table 4). Conversely, all plants from the interploid crosses produced seeds. In both plant groups, the seed germination rates oscillated from medium to high values (Table 4). Most seedlings presented two cotyledons, although seedlings that originated from apomictic seeds showed a higher percentage of anomalies such as pleiocotyly (one, two, three, or four cotyledons) and polyembryony (i.e., the formation of more than one embryo per ovule) than those originated from hybrids’ seeds (Table 5). Seedlings with more than two cotyledons or polyembryony derived from hybrid (*n* = 17) or apomictic (*n* = 255) plants revealed no differences in ploidy in relation to parental plants (Figure S2).

## 3. Discussion

The effects of hybridization, polyploidy (eu-, aneuploidy), and apomixis appear to have all contributed to shape the radiation of the genus *Limonium* [1,2,4,7,24,25,26,27,28,29]. Our investigation on plants obtained after controlled hand pollination crosses, using four species of *Limonium* from different species complexes, represents an attempt to evaluate the influence of the *Limonium* polymorphic sexual system and insect behavior in their reproductive outcomes inferred from an open-pollination experiment. Our analyses revealed several novel findings. (1) Crosses between diploid species produced fertile and infertile hybrids with both the maternal and paternal pollen-stigma combinations and inflorescence types, and some of them presented intermediate inflorescence morphotypes. (2) Crosses between diploids and tetraploids originated fertile plants with the maternal morphotype only but with high levels of inviable pollen. (3) Pollen transport (A and/or B) by distinct insect visitors was observed in all plants, independently of pollen fertility and plants’ reproductive modes, sexual or apomixis. The findings support that apomixis is a reproductive barrier between the diploid and the tetraploid species. Nonetheless, since we carried out an open-pollination experiment, and there are species in the *L. binervosum* complex that seem to be facultative apomicts [23], rare hybridization events through reproductive interference mediated through cross-fertilization of sexuals by apomicts could occur.

A previous study using pollen-stigma polymorphic *Limonium* plants as progenitors revealed diverse diploid hybrids arising from sexual reproduction in homoploid crosses and tetraploid plants with the maternal phenotype produced by apomixis in heteroploid crosses [4]. Here, we found that diploid hybrids globally have similar inflorescence types and self-incompatible combinations (i.e., pollen A/*cob*-like stigma or pollen B/papillate stigma) like parental plants [4]. Two of the hybrids presented inflorescence morphologies like *L. binervosum* and were tetraploid. These unexpected phenotypes could hypothetically be due to reproductive interferences mediated by cross-pollination between plants since apomicts produced both viable and inviable pollen grains. Asymmetric reproductive interference in favor of apomicts was found in the case of cross-fertilization by cytotypes of differing ploidy, involving sexuals and apomicts in *Potentilla puberula* (Rosaceae) [30]. In mixed populations in the field, apparently, reproductive interferences of *P. puberula* sexuals by apomicts appear to be negligible [31]. In the case of the species studied here, mixed-ploidy populations of sexual diploids of *L. ovalifolium* complex and apomicts tetraploids of *L. binervosum* complex rarely occur [12,24]. Morphological, cytogenetic, and genetic studies using cpDNA (Chloroplast DNA) markers and MSAP (Methylation Sensitive Amplified Polymorphism) markers demonstrated a differentiation between diploid and tetraploid taxa of the studied sexual and asexual complexes [7,25,29]. Although cpDNA analyses revealed species-specific lineages, suprahaplotypes (closely related haplotypes) were found to be shared among taxa. Even if there is transference of pollen from apomicts to sexuals in the open-pollination experiment here studied, it is not known whether this phenomenon occurs in the field.

In apomicts the inflorescences and pollen-stigma combination are of maternal type (i.e., A/*cob*-like stigma), with exception of one plant that was self-compatible (i.e., pollen A/papillate stigma). It could be hypothesized that this latter finding could result from the breakdown of the self-incompatibility system brought about by cross-pollination. Alternatively, this result could be interpreted as the result of a (rare) hybridization event. For example, in the apomictic *Ranunculus auricomus* (Ranunculaceae) polyploid complex both diploid sexual taxa and their F_1_ hybrids are completely self-incompatible, but breakdown of self-incompatibility was observed in the rather widespread allohexaploid apomicts (a phenomenon known as mentor effects) [32]. Mentor effects were also found in diploid *Hieracium alpinum* (Compositae) under the influence of foreign pollen from another species of this genus during a series of crossing experiments and after cross-fertilization of sexuals and apomicts of *P. puberulla* [30,33].

The diploid hybrids display medium to high pollen viability similarly to their parental plants (Figure S1). Both diploid hybrids and tetraploid apomicts produce pollen grains with either more or less than three *colpi*, although the most frequent pollen grain type in both plant groups present three *colpi* and are viable. At least in some of the crosses, the low level of pollen fertility found in the apomictic progeny contrasts with that of their apomictic mother plants that show high pollen fertility (Figure S1). This unexpected result could be explained that during the flowering period the pollen viability of some of the apomicts decreased gradually and more rapidly than in sexual hybrids, i.e., the pollen had low longevity. At least one species of the *L. binervosum* complex (*L. *transwalianum)** is a facultative apomict [23]. In this case, the pollen of this species may be functional at least for endosperm formation and/or eventually it could be transferred to sexuals in sympatric populations. Another apomict tetraploid from this complex is *L. multiflorum* that forms irregular, abnormal microspores with a collapsed morphology without the typical exine patterns, which are inviable [12]. All the plants from the apomictically established progeny form heterogeneous pollen probably related with residual male fertility due to meiotic disturbance caused by nonreduction via meiotic restitution as found in other *Limonium* apomicts (e.g., triploid *L. algarvense* Erben) [34].

Pollen viability levels seem to be important to determine flower visitation in some plant families like the Melastomataceae, since apomictic plants with no viable pollen or with pollen with low viability are not visited by pollinators [35]. Here, pollinators belonging to Lepidoptera, Hymenoptera, Diptera, Coleoptera, and Heteroptera orders visited flowering plants and carried A or B pollen on their bodies, irrespective of plants’ pollen viability and reproductive modes (i.e., sexual or apomixis). Field studies reveal that *Limonium* flowers provide floral resources (pollen and nectar) to a diverse range and number of insects from different orders, including Diptera, Hemiptera, Heteroptera, Hymenoptera, and Lepidoptera (e.g., [15,35,36]). The greatest number of specimens visiting inflorescences and carrying *Limonium*-type pollen [9,10,37,38] on their bodies are from the species *C. coriacana* (18% with pollen) and *Tapinoma* sp. (9% with pollen). Further, a high level of *C. coriacana* pupae and *exuviae* was detected in both *Limonium* stored seeds from experimental collections (this study) and natural populations (Sofia I. R. Conceição and A. D. Caperta, unpublished study). *Limonium barceloi* seeds from field plants are predated by insect (moth) larvae but the identity of this moth was not yet reported [15]. In fact, all stages of *Clepsis coriacana* (egg, larvae, pupae, adult) development are present in association with various organs of *Limonium* plants and use these plants as a food source (larvae feed on leaves and adults feed on pollen) and as a habitat (eggs are placed on leaves and larvae use leaves and flowers to pupate). Although *C. coriacana* being probably an exotic species, which was recently reported in Portugal [39] and for the first time in Europe (Gibraltar, in 2006) [40], the fact that it completes its life cycle in *Limonium* plants is strong evidence that this phytophagous insect lives associated with these plant hosts and actively participates in their pollination. Since *C. coriacana* has never been observed in association with other plants outside the greenhouse, it was likely transported along with specimens or parts of *Limonium* (i.e., leaves, inflorescences, seeds) from the field (e.g., south of Portugal and Morocco) and found favorable conditions for its establishment (i.e., climatic, plant hosts) in the greenhouses of Instituto Superior de Agronomia, Lisbon, Portugal. The prevalence of *Limonium* flowers’ visits by *C. coriacana* supports its high dependence of these host plants for completing its life cycle, sustaining that this moth is an effective pollinator of *Limonium* plants.

In open cross-pollination experiment carried out in this study, it was found that the fertility of the diploid hybrids is variable, and not all plants produced seeds. Plants that did not produce seeds were from both intra- and interspecific crosses and had either combination type A or B. It could be hypothesized that in this latter case insect visits probably were not effective in pollination with the reciprocal pollen morph, as all hybrid plants were self-incompatible. However, the hybrids that show fertile inflorescences produced seeds with medium to high seed germination rates. Since these hybrids and parental plants are closely genetically related [4], severe meiotic disturbance leading to male and/or female infertility is not expected. Therefore, the reproductive barriers between these species/plants seem to be weak or inexistent. This might explain the finding that the diploid hybrid plants exhibit very few seed developmental anomalies like pleicotyly (more than two cotyledons) and polyembryony (i.e., the formation of more than one embryo per ovule) [41,42]. By contrast, all the apomicts produced seeds, even with low levels of pollen fertility, that apparently did not block endosperm formation. The progeny resulting of the apomictic seed development of maternal plants show various developmental seed anomalies, eventually produced by asynchronous and ectopic expression of several developmental pathways due to the presence of multiple genomes in polyploid plants [42]. Nonetheless, whether the tetraploid apomicts arise from autopolyploidy or allopolyploidy and which genomes are involved in their genesis has not yet been investigated, as far as we know. Finally, plants from each apomict progeny form fertile seeds that originate anomalous seedling (pleiocotyls, polyembryonic seedlings).

In conclusion, this study demonstrates that there are weak reproductive barriers between two of the species of the *L. ovalifolium* complex, since in interspecific crosses fertile hybrids were formed, at least in experimental, controlled crosses. Pollen vectors like insects appear to be important in cross-pollination of such species, as some of the self-incompatible hybrids did not produce seeds. Contrastingly, apomixis is a reproductive barrier between the diploid and tetraploid plants representing different complexes, since heteroploid crosses (apomictic tetraploid × sexual diploid) failed to produce true hybrids and the progeny arose via apomixis. Unfortunately, we were unable to make the reciprocal cross (sexual diploid × apomictic tetraploid) with the same pair of individuals that hypothetically could result in true hybrid progeny through male gametophyte from (pollen) apomicts. At least, tetraploid *Limonium transwallianum* belonging to the *L. binervosum* complex is a facultative apomict species [23]. Thus, we cannot exclude that, in the case of sympatry of diploid and tetraploid species, rare hybridization events via reproductive interferences of apomicts in sexual plants may occur.

## 4. Materials and Methods

### 4.1. Plant Material and Growth Conditions

The plants investigated in this study were originated in a controlled pollination ex situ experiment carried out in spring 2016 in the greenhouses of Instituto Superior de Agronomia (ISA), University of Lisbon, Portugal (Figure 1). Parental plants were established from seeds collected from natural populations. A total of 20 plants representative of diploid species *L. ovalifolium* and *L. nydeggeri* (2*n* = 2x = 16 chromosomes) and tetraploid species *L. binervosum* and *L. dodartii* (2*n* = 4x = 35, 36 chromosomes) [7,12], respectively, were used in controlled homoploid and interploid crosses [4] (Figure 1a). Six diploid *L. ovalifolium* and *L. nydeggeri* self-incompatible plants with combination A or B (Figure 1a,b) were utilized in both intra- and interspecific homoploid (diploid × diploid) reciprocal crosses. In heteroploid crosses (tetraploid × diploid) five *L. binervosum* and three *L. dodartii* tetraploid plants with combination A were used. Reciprocal crosses using tetraploid plants were not possible to conduct, as until now we were unable to find mother plants with combination B in the field. No flower emasculation prior to anther dehiscence was performed in inflorescences as the plants were self-incompatible [4,7]. The resulting plants from homoploid and heteroploid crosses, respectively, hybrids and apomicts (Figure 1c), were assessed using morphological reproductive and flow cytometry methodologies, and progeny analyses were made using nuclear ribosomal DNA sequences [4,7].

The present reproductive study is based on the hybrids (*n* = 36) and apomicts (*n* = 15) obtained in the above-explained homoploid and interploid crosses [4]. Plants were cultivated within a semi-closed greenhouse at ISA/UL and grown in pots (5-cm diameter) using a commercial substrate (Siro, SIRO, Mira, Portugal).

### 4.2. Inflorescence Phenotype, Floral Heteromorphisms, and Pollen Viability

To determine the inflorescence morphotypes (scapes’ morphology) of hybrids and apomicts, and if these corresponded to morphotypes similar to their parental plants (i.e., *L. ovalifolium*, *L. nydeggeri*, *L. binervosum*, *L. dodartii*) or new morphotypes, offspring plants were visually inspected during the flowering season (May–July 2019).

A total of three fresh flowers per plant was used for floral heteromorphism determinations, following [4,7]. Pollen and stigmas were dissected and covered with a drop of water and preparations were observed by light microscopy (Leitz HM-LUX 3), LEICA, Portugal), with ×400 magnification. Plants were classified as having either A, B, C, or D pollen-stigma combinations (Figure 1, [7]).

For pollen viability estimations, from each cross, the pollen viability was counted considering all plants of a particular crossing (Figure S1). Fresh anthers from mature flowers at anthesis were collected, squashed, and the pollen spread onto the microscope slide. The grains were stained using Alexander’s stain [43] under a coverslip and observed under light microscopy (Leitz HM-LUX 3). Total pollen viability estimates were performed by one person using three flowers per plant and counted with a 40× objective.

### 4.3. Open Pollination Experiment and Floral Visitors

Parental plants as well as each hybrid and apomictic plants originated from the above controlled crosses (Figure 1) were subjected to an open cross-pollination experiment and allowed to be visited by insects in the semi-closed greenhouse. Censuses were taken of insect visitors that regularly visited plants. Around 270 flowering stems were observed for 20 counts of 1 h, making a total of 20 h of observations from May to July 2019. Observations were carried out between 10:00 h and 19:00 h, during the period when insect adult flight abundance is greatest. Only the visitors touching reproductive parts of the flower were included in this study, which were then collected and taken to the laboratory for identification of insects’ species and type of pollen grains carried in their body. Only the most abundantly collected specimens were identified at genus and species level, namely, Lepidoptera and Hymenoptera, as in [44,45]. Pollen grains (A or B types) transported by the different visitors were photographed using the camera ProgRes CT5 (Jenoptik Optical Systems, Jena, Germany) combined with the stereoscopic microscope stereoscopic microscope Mei-jiTechno model PKL-1Stand (Japan) (70×).

### 4.4. Seed Germination and Seedlings’ Phenotype Evaluation

Nine weeks after the open pollination experiment the seeds from hybrid and apomict plants, above mentioned, were harvested. Seeds were placed in Petri dishes with water-soaked filter paper in a growth chamber (Rumed), with controlled light and temperature conditions, at 18 h light/6 h dark, at 25 °C [12]. After two months, the seedlings were transferred to planting pots with commercial substrate (Siro) and kept in the semi-closed greenhouse. Each seedling was visually inspected to determine its phenotype, whether it fit the appearance of parental plants or if it presented a new morphology.

### 4.5. Genome Size and DNA Ploidy Estimations in Seedlings

The progeny of the open pollination experiment with atypical cotyledon number (>2; pleiocotyly) and polyembryonic (>1 embryo in one seed) seedlings were evaluated individually by flow cytometry to verify if their ploidy levels differed from their parental plants. Nuclei were isolated following the procedure of Galbraith et al., 1983 [46], in which 0.5 cm^2^ of fresh leaf tissue of each sample was chopped with a razor blade, simultaneously with 0.5 cm^2^ of fresh leaf tissue of the internal reference standard, in a Petri dish containing 1 mL of Woody Plant Buffer (WPB) [47]. As internal standard, *Pisum sativum* ‘Ctirad’ (2C = 9.0 pg) or *Secale cereale* ’Dankovske’ (2C = 16.19 pg) [48] was utilized. The suspension was filtered through a 50-µm mesh nylon filter, and propidium iodide (50 µg/mL) was added to stain the DNA. To avoid staining of double-stranded RNA, 50 µg.mL-1 of RNAse (Fluka, Buchs, Switzerland) were also added to the suspension. After a 5-min incubation period, the samples were analyzed in a Partec CyFlow Space flow cytometer (Partec GmbH, Görlitz, Germany) equipped with a green, solid-state laser (Cobolt Samba 532 nm, operating at 30 mW; Cobolt, Stockholm, Sweden), used to measure the relative fluorescence of stained nuclei. Results were obtained using PARTEC FLOMAX software (v. 2.9) (Sysmex Partec GmbH, Görlitz, Germany). About 5000–5500 nuclei per sample were examined.

The DNA-ploidy level was inferred as a relative position of the sample G1 peak to that of the internal standard. The value of genome size in mass units (2C in pg; *sensu* [49]) was obtained for each individual analyzed, using the following equation: *Limonium* 2C nuclear DNA content (pg) = (*Limonium* G_1_ peak mean/reference standard G_1_ peak mean) * genome size of the reference standard.

### 4.6. Statistical Analyses

Three binomial response variables were studied: (1) the pollen-stigma combination data, which were defined as the number of plants that presented pollen-stigma combination A (successes) out of the total number of plants analyzed; (2) the *colpi* number of pollen grains, which was defined as the number of grains with *3 colpi* (successes) out of the total number of pollen grains analyzed; and (3) cotyledons’ number data, which were defined as the number of seedlings with two cotyledons (successes) out of the total number of seedlings. In all cases, a generalized, linear, mixed model with a logit link function was fitted, considering one fixed effects’ factor “reproductive modes”, with two levels (sexual and apomixis), considering all crosses (five from sexual reproduction and four from apomixis reproduction). A likelihood ratio test, to compare reduced and full model, was conducted to test the effects of the mode of reproduction in the response variable studied. Data analysis was performed in R studio version 1.2.1335 software using the glm function.

## Figures and Tables

**Table 1:** Pollen-stigma combinations and inflorescence phenotypes of investigated plants. These plants were produced in homoploid (diploid × diploid) and heteroploid (tetraploid × diploid) experimental crosses carried out in [4]. * Pollen-stigma combinations followed [7]: A: coarsely reticulate exine (A pollen)/*cob*-like stigma; B: finely reticulate exine (B pollen)/papillate stigma; and C: coarsely reticulate exine (A pollen)/papillate stigma. A and B represent self-incompatible combinations (self-sterile) and C is a self-compatible (self-fertile) combination. No individuals with the self-compatible combination D (microreticulate pollen/*cob*-like stigma) were reported. The intermediate inflorescence morphotype combines characteristics of scapes from both *Limonium ovalifolium* and *L. nydeggeri*. Symbols: ♀—female recipient; ♂—male donor.

Crosses	Parental Plants	Pollen-Stigma Combinations *			Inflorescence Morphotypes					Total
		A	B	C	*L. nydeggeri*	Intermediate	*L. ovalifolium*	*L. binervosum*	*L. dodartii*	
Homoploid	A♀ × B♂/B♀ × A♂	19	20	0	8	9	18	1	0	36
Heteroploid	A♀ × B♂	14	0	1	0	0	0	14	1	15

**Table 2:** The probability and the likelihood ratio test (LRT) and respective *p*-value for the traits studied.

Trait	Type of Reproduction	Probability	LRT Statistic Value (*p*-Value)
*Colpi* number	Sexual	0.99	580.85 (<2.2 × 10^−16^)
	Apomixis	0.34	
Cotyledons number	Sexual	0.97	45.57 (1.477 × 10^−11^)
	Apomixis	0.81	
Pollen-stigma combination	Sexual	0.59	16.61 (4.59 × 10^−5^)
	Apomixis	1.00	

**Table 3:** Number of pollen grain types and percentage of pollen viability observed in plants obtained in homoploid (diploid × diploid) and heteroploid (tetraploid × diploid) crosses. The data on species and on experimental crosses were derived from [4]. The species marked with an asterisk (*) reproduced by apomixis [4]. N represents the number of individuals/cross analyzed. Symbols: ♀—female recipient; ♂—male donor.

N	Cross (#) ♀ × ♂	Viable Pollen with #*colpi*					Inviable Pollen with #*colpi*					Pollen Fertility	*N*
		1	2	3	4	5	1	2	3	4	5	(%)	
Homoploid	(1) *L. ovalifolium* × *L. ovalifolium*	0	0	2507	0	0	1	0	349	2	0	87.7	6
	(2) *L. nydeggeri* × *L. ovalifolium*	0	0	2709	0	0	0	2	480	0	0	84.9	8
	(3) *L. nydeggeri* × *L. ovalifolium*	0	0	548	17	0	0	5	123	35	12	76.4	3
	(4) *L. ovalifolium* × *L. ovalifolium*	0	0	2386	0	0	0	1	556	0	0	81.1	7
	(5) *L. nydeggeri* × *L. ovalifolium*	1	1	2547	2	36	18	13	213	85	193	83.2	7
Heteroploid	(8) *L. binervosum* * × *L. nydeggeri*	0	0	26	21	9	32	132	1150	250	27	3.4	4
	(11) *L. binervosum* * × *L. nydeggeri*	0	0	0	0	0	8	31	317	91	0	0	6
	(12) *L. binervosum* * × *L. nydeggeri*	0	0	14	20	21	32	63	1593	442	53	2.5	4
	(13) *L. dodartii* * × *L. nydeggeri*	0	0	0	0	1	6	23	300	115	9	0.2	1

**Table 4:** Number of seeds and rates of seed germination from open cross-pollination experiment of plants originated in homoploid (diploid × diploid) and heteroploid (tetraploid × diploid) crosses. The data on species and on experimental crosses were derived from [4]. The species marked with an asterisk (*) reproduce by apomixis [4]. The number given in parentheses (#) represents the number of germinated seeds. Symbols: ♀—female recipient; ♂—male donor.

	Cross (#) ♀ × ♂	Number of Seeds	% Seed Germination (#)
Homoploid	(1) *L. ovalifolium* × *L. ovalifolium*	1	100 (1)
	(2) *L. nydeggeri* × *L. ovalifolium*	129	62 (80)
	(3) *L. nydeggeri* × *L. ovalifolium*	149	90 (134)
	(4) *L. ovalifolium* × *L. ovalifolium*	84	81 (68)
	(5) *L. nydeggeri* × *L. ovalifolium*	107	84 (90)
Heteroploid	(8) *L. binervosum* * × *L. nydeggeri*	257	82 (210)
	(11) *L. binervosum* * × *L. nydeggeri*	320	33 (106)
	(12) *L. binervosum* * × *L. nydeggeri*	170	65 (110)
	(13) *L. dodartii* * × *L. nydeggeri*	100	94 (94)

**Table 5:** Percent of seedlings with certain morphotypes and polyembryony from open cross-pollination experiment of plants originated in homoploid (diploid × diploid) and heteroploid (tetraploid × diploid) crosses. The data on species and on experimental crosses were derived from [4]. The species marked with an asterisk (*) reproduce by apomixis [4]. The number given in parentheses represents the number of seedlings. Symbols: ♀—female recipient; ♂—male donor.

	Cross (#) ♀ × ♂	Seedling Morphotypes #Cotyledons				Poly-Embryony
		1	2	3	4	
Homoploid	(1) *L. ovalifolium* × *L. ovalifolium*	0	100 (1)	0	0	0
	(2) *L. nydeggeri* × *L. ovalifolium*	0	100 (80)	0	0	0
	(3) *L. nydeggeri* × *L. ovalifolium*	0	90 (120)	6 (8)	0	4 (6)
	(4) *L. ovalifolium* × *L. ovalifolium*	0	97 (66)	3 (2)	0	0
	(5) *L. nydeggeri* × *L. ovalifolium*	1 (1)	99 (89)	0	0	0
Heteroploid	(8) *L. binervosum* * × *L. nydeggeri*	0	83 (172)	5 (10)	0	12 (26)
	(11) *L. binervosum* * × *L. nydeggeri*	0	67 (71)	3 (3)	2 (2)	28 (30)
	(12) *L. binervosum* * × *L. nydeggeri*	0	77 (85)	3 (3)	4 (4)	16 (18)
	(13) *L. dodartii* * × *L. nydeggeri*	0	97 (91)	1 (1)	0	2 (2)

## Data Availability

Not applicable.

## Supplementary Materials

The following are available online at https://www.mdpi.com/2223-7747/10/1/169/s1. Figure S1: Pollen viability observed in parental plants and progeny obtained in homoploid (diploid × diploid) and heteroploid (tetraploid × diploid) controlled crosses. Figure S2: Genome size estimations of seedlings from open cross-pollination experiment of plants originated in homoploid (diploid × diploid) and heteroploid (tetraploid × diploid) crosses.
