# Supplementary material for: Reproductive Output and Insect Behavior in Hybrids and Apomicts from Limonium ovalifolium and L. binervosum Complexes (Plumbaginaceae) in an Open Cross-Pollination Experiment
Source: Plants (Basel). 2021 Jan 17;10(1):169. doi: 10.3390/plants10010169 (PMC7830651; doi:10.3390/plants10010169)

Article

Reproductive Output and Insect Behavior in Hybrids and Apomicts from *Limonium ovalifolium* and *L. binervosum* Complexes (Plumbaginaceae) in an Open Cross-Pollination Experiment

Sofia I. R. Conceição [1], Joana Fernandes [1], Elsa Borges da Silva [2] and Ana D. Caperta [1,*]

[1] Linking Landscape, Environment, Agriculture and Food (LEAF), Instituto Superior de Agronomia (ISA), Universidade de Lisboa, Tapada da Ajuda, 1349-017 Lisboa, Portugal; sofia-conceicao@hotmail.com (S.I.R.C.); joanabnf@hotmail.com (J.F.)

[2] Forest Research Centre (CEF), Instituto Superior de Agronomia (ISA), Universidade de Lisboa, Tapada da Ajuda, 1349-017 Lisboa, Portugal; elsasilva@isa.ulisboa.pt

*Correspondence: anadelaunay@isa.ulisboa.pt

Figure 1. Pollen viability observed in parental plants and progeny obtained in homoploid (diploid x diploid) and heteroploid (tetraploid x diploid) controlled crosses. The data on species and on experimental crosses were derived from [4]. The pollen viability of progenitor plants used as female recipients (♀) and male donors (♂) in [4] and of plants produced by these crosses are represented. From each cross, the pollen viability was counted considering all plants of the particular crossing experiment.

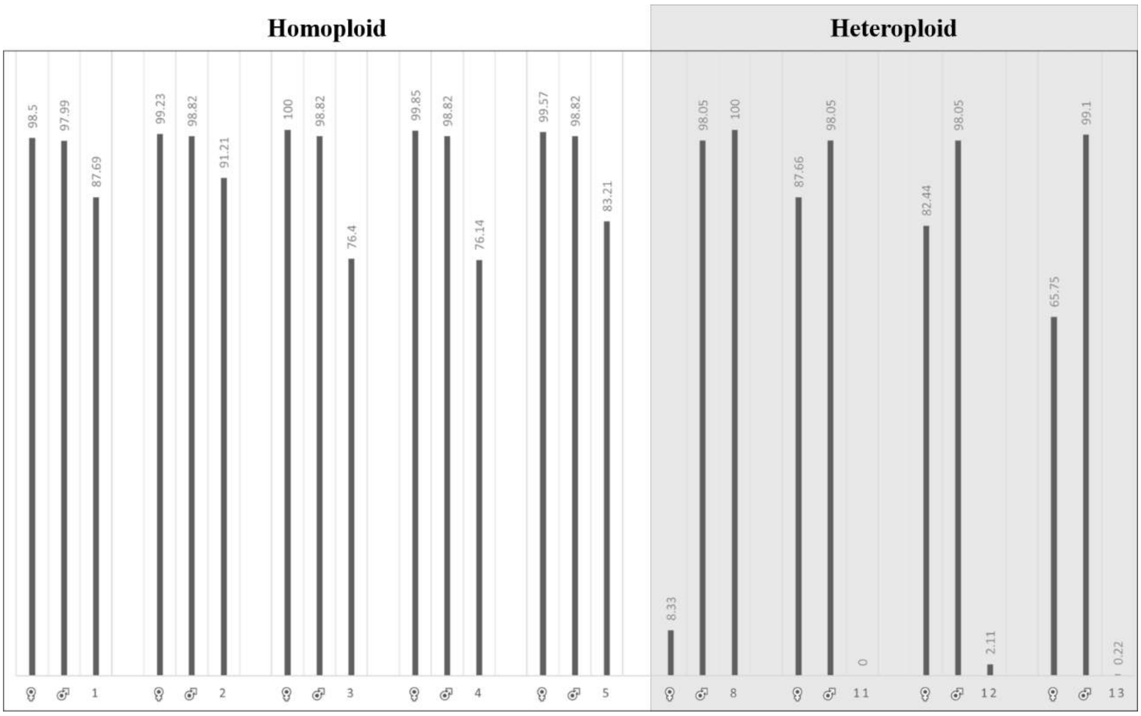

Figure S2. Genome size estimations of atypical seedlings from open-cross pollination experiment of plants originated in homoploid (diploid x diploid) and heteroploid (tetraploid x diploid) crosses. The data on species and on experimental crosses were derived from [4]. The atypical seedlings include tricotyls, tetracotyls and polyembryonic seeds. Crosses one, two and five were not used in this analysis since they did not produce seedlings with the referred anomalies.

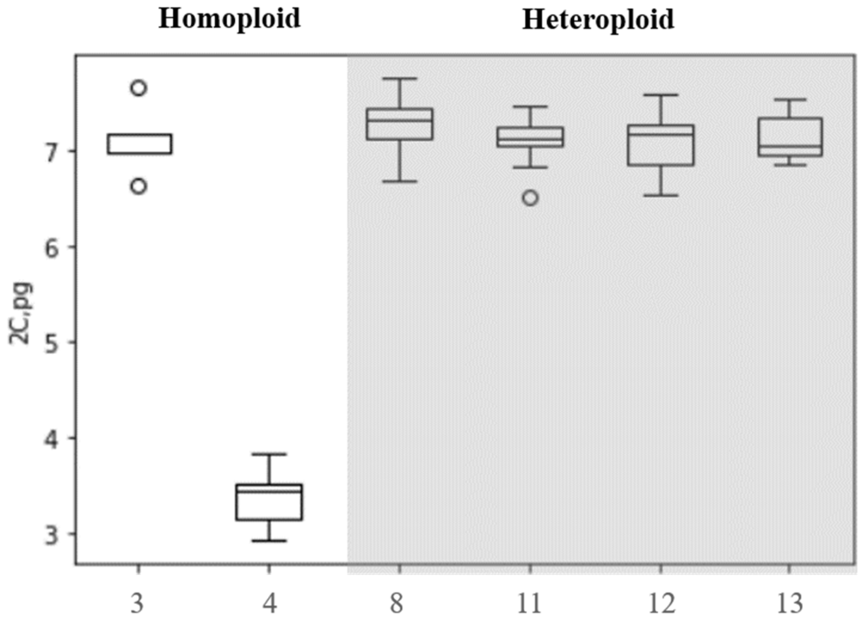

Supplement: Supplementary file 1 [file plants-10-00169-s001.pdf]
